# Radiographic Prevalence of Anatomical Variations of the Ventral Lamina of the Sixth Cervical Vertebra, C6/C7 Articular Process Joint Modelling and Competition Outcomes in Warmblood Sport Horses

**DOI:** 10.3390/ani16030424

**Published:** 2026-01-29

**Authors:** Teresa Strootmann, Vanessa G. Peter, Jens Körner

**Affiliations:** 1 Hanse Equine Clinic, 27419 Sittensen, Germany; jens.koerner@hanseklinik.com; 2 Rossdales Equine Diagnostic Centre, Newmarket CB8 7NN, UK

**Keywords:** equine, cervical spine, ECVM, ECCMV, C6 morphology, facet joint, competition performance, osteoarthritis

## Abstract

Anatomical variations of the caudal ventral lamina of the sixth cervical vertebra (C6), referred to as equine caudal cervical morphologic variations (ECCMV), and radiographic modelling of the caudal cervical articular process joints (APJ) are commonly identified in sport horses, yet their interrelationship and relevance for athletic performance remain controversial. This retrospective observational study aimed to determine the prevalence of ECCMV and APJ modelling in a clinically sound warmblood sport horse population, to evaluate a potential association and to assess if ECCMV affects competition performances. Records of 200 warmbloods, presented for pre-purchase examination between 2020 and 2024 were reviewed: the predominant breed was Hanoverian (*n* = 131), followed by Oldenburg (*n* = 27) and Holsteiner (*n* = 22), including 127 geldings, 61 mares and 12 stallions with a median age of 5. ECCMV was diagnosed on latero-lateral radiographs if the caudal ventral lamina of C6 was uni- or bilaterally absent, and APJ modelling at C6/C7 was graded as either normal or mild or moderate to severe. Competition placings and lifetime earnings of each horse were collected from the German National Equestrian Federation database. ECCMV was found in 30% of horses and APJ modelling in 32.5% (mild 26.5%, moderate/severe 6%). There was no significant association between the presence of ECCMV, APJ alterations and competition results, but horses with ECCMV were less likely to exhibit radiographic changes in the adjacent APJ compared to those with a normal C6 morphology (23.3% vs. 36.4%). In conclusion, ECCMV is a common anatomical variation in clinically sound warmbloods, neither predisposing them to APJ modelling nor limiting athletic performance.

## Simple Summary

Recently, anatomical variations in the shape of the sixth cervical vertebra (C6) have received considerable attention both among equine practitioners and in the equine community. The clinical significance of this finding, referred to as equine caudal cervical morphologic variation (ECCMV), is still controversial. The same applies for alterations in the radiographic appearance of articular process joints (APJ), connecting adjacent vertebrae. Such changes are frequently detected, including in horses without clinical signs. In this study, radiographs of C6 and competition records of 200 clinically sound warmblood sport horses were evaluated. ECCMV and APJ alterations were present in approximately one third of the horses. Statistics yielded no significant association between these findings, but horses with ECCMV were less likely to exhibit radiographic changes in the adjacent APJ. Competition results did not differ between horses with and without ECCMV. These findings suggest that ECCMV is a common anatomical characteristic rather than a disease and does not appear to limit athletic performance in warmbloods.

## 1. Introduction

Although the existence of equine vertebral morphologic variations has been reported in the 20th century already [1], more recently, great attention is being paid to differing anatomical characteristics of the sixth and subsequently seventh cervical vertebrae as well as to aplasia, hypoplasia or synostosis of the first two ribs [2,3,4,5,6,7].

A unique feature of the sixth cervical vertebra (C6) is the so-called ventral lamina or caudal ventral tubercle, a large ventral expansion of the left and right transverse processes. Morphologic variations like the symmetric (bilateral) or asymmetric (unilateral) absence of the caudal ventral lamina and its possible transposition onto the ventral surface of the seventh cervical vertebra (C7) are considered to be congenital [5,7]. The extent of absence per tubercle varies and has been thoroughly investigated and documented by different grading systems [5,6].

The prevalence of these equine caudal cervical morphologic variations (ECCMV) ranges from 13% in a mixed-breed population [8] up to 38% in thoroughbreds [9]. To date, there are several studies on the association of ECCMV with biomechanical and neurological changes potentially leading to neck pain and decreased cervical range of motion, ataxia, lameness or abnormal behaviour [5,6,10,11,12]. Despite ongoing research, a clear consensus on the clinical significance and a potential impact on equine performance has not yet been reached.

A comparable ambiguity exists regarding the interpretation of radiographic evidence of articular process joint modelling (APM). Enlargement of the articular processes due to osteophyte formation, degenerative joint disease or biomechanical stress can lead to narrowing of the intervertebral foramen and spinal nerve root compression [13,14,15,16]. It is reported that severe modelling of the APJ can result in proprioceptive deficits, forelimb lameness and poor performance such as stumbling, neck pain and stiffness [14,17]. However, radiographic signs of APM in the caudal vertebral region are a relatively common finding in both symptomatic and asymptomatic horses, with prevalences of up to 67% in actively performing sport horses [18], which makes clinical implications appear controversial [19,20,21,22].

Most of the studies reporting on the presence of ECCMV have focused on clinically affected horses or populations presented for diagnostic evaluation. Consequently, ECCMV is frequently interpreted as a potentially pathological finding, despite limited evidence regarding functional ramifications. Data on the prevalence of ECCMV in clinically sound horses, its association with APM and its potential impact on athletic performance remain scarce. Due to the considerably varying and with a focus on German warmblood breeds partially missing data on these two conditions, this study aimed to (1) assess the prevalence of ECCMV and APM in a clinically sound adult warmblood sport horse population, (2) to track and compare competition performances of horses with and without ECCMV and (3) to investigate a potential association between the occurrence of ECCMV and APM.

By focusing on a non-clinical sport horse population and incorporating standardized radiographic assessment together with objective competition performance data, this study addresses relevant gaps in the current literature and contributes to a more differentiated interpretation of ECCMV as an anatomical variation rather than an inherently pathological condition.

In the scope of this study, ECCMV is defined as partial or complete uni- or bilateral absence of the ventral laminar part of the transverse process of C6 in combination with or without a ventral protuberance at the transverse process of C7.

Based on observations in clinical practice, the authors hypothesized that performance outcomes for horses with and without ECCMV do not differ significantly and that ECCMV is negatively correlated with APM.

## 2. Materials and Methods

### 2.1. Study Design

The study followed a retrospective and observational design.

#### 2.1.1. Study Population

Records of all horses presented for pre-purchase examination at Hanse Equine Hospital in Sittensen, northern Germany, between 2020 and 2024 were reviewed. Signalment including breed, age and sex were documented based on general adspection of the horse, dental age estimation, passport data and reading of the implanted microchip. Every pre-purchase examination firstly comprised a comprehensive clinical examination including mobilization of the neck. Specific palpation of anatomical structures of the caudal cervical region was not part of this standard pre-purchase approach. Secondly, the gait assessment was carried out on a straight line and circle on hard ground in-hand and on soft ground lunged. Routine screening flexion tests followed. The subsequent radiographic examination included at minimum the standard set of 18 radiographs of the limbs recommended by the German Society for Equine Medicine, supplemented by additional views upon the client’s request.

Inclusion criteria for this study were as follows: Warmblood breed, pre-purchase examined with generally accepted abnormalities considered as low risk for the development of lameness in accordance with the German radiographic guidelines for horse sales (issue 2018, by German Equine Veterinary Association GEVA/GPM), soundness and the availability of latero-lateral radiographs of the cervical spine permitting adequate evaluation of C6 and C7.

The same examiner (Dipl. ECVSMR/ACVSMR) was present at all procedures including the clinical examination and radiography.

#### 2.1.2. Radiographic Assessment

Radiographs were obtained using digital radiography systems (Gierth Scope Ultralight 820C, Gierth X-Ray international, Riesa, Germany), and images were processed and reviewed using medical image management software (Horos, Version 4.0; Horos Project, Annapolis, MD, USA) on a medical diagnostic imaging display screen.

**Figure 1:**
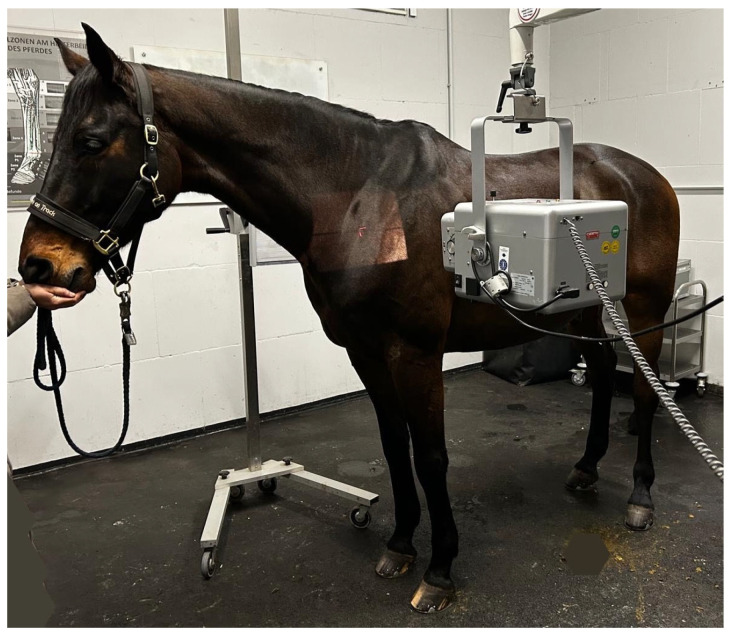
Latero-lateral left to right radiographic image acquisition. Positioning of the horse with its head slightly elevated and both forelimbs shifted caudally; film-focus distance 100 cm.

For image capture, intravenous sedation was administered using a combination of Detomidine Hydrochloride (Domidine 10 mg/mL; Dechra, Aulendorf, Germany) and Butorphanol (Torphadine 10mg/mL, Dechra). Each horse was positioned with all legs square and perpendicular to the ground, with the cervical spine held in a straight alignment; manual stabilization of the head was applied when necessary. To enhance visualization of the caudal cervical region, particularly C6 and C7, and to avoid superimposition with the scapula, the head was carefully elevated and both forelimbs shifted caudally. Left to right lateral views were taken with exposure settings ranging from 72 to 80 kV and 2.4 to 3.8 mAs, and a film-focus distance of 100 cm was adhered to. A minimum set of six radiographs was taken to capture the entire cervical spine. A photo of the image capturing procedure is provided in Figure 1.

Radiographic evaluation was performed independently by a European College of Veterinary Diagnostic Imaging (ECVDI) board-certified veterinary radiologist and a European/American College of Veterinary Sports Medicine and Rehabilitation ECVSMR/ACVSMR board-certified veterinary orthopedic specialist (Dipl. ECVSMR/ACVSMR). In the few cases of inconsistent reviews concerning ECCMV, the respective radiographs were reassessed and a consensus reached. Inter-observer reliability for categorical data (ECCMV presence: yes/no) was quantified using Cohen’s kappa coefficient (kappa values were interpreted according to Landis and Koch: <0.00 poor, 0.00–0.20 slight, 0.21–0.40 fair, 0.41–0.60 moderate, 0.61–0.80 substantial and 0.81–1.00 almost perfect agreement).

For interpretation, all images were aligned cranial to the left and caudal to the right.

**Figure 2:**
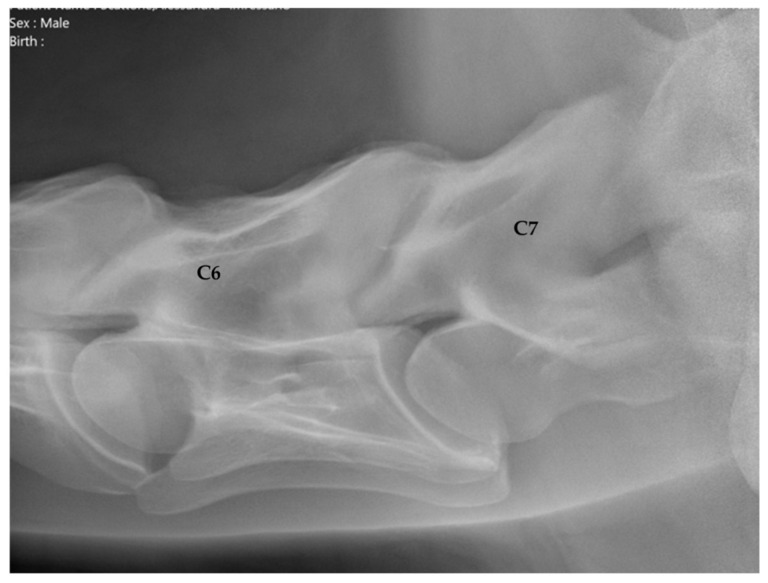
Latero-lateral radiographic image of C6 with a complete ventral lamina of a 5-year-old Hanoverian gelding. Cranial is to the left.

**Figure 3:**
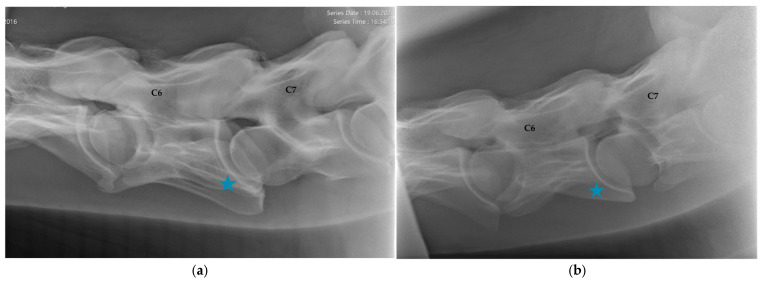
(**a**) Latero-lateral radiographic image of C6 with a unilaterally absent ventral lamina, marked with a blue asterisk, (equine caudal cervical morphologic variation, ECCMV) of an 8-year-old Oldenburg mare. C7 shows a vestigial ventral lamina on the caudoventral aspect of the vertebral body. Cranial is to the left. (**b**) Latero-lateral radiographic image of C6 with a bilaterally absent ventral lamina, marked with a blue asterisk, and its transposition onto C7 (equine caudal cervical morphologic variation, ECCMV), of a 6-year-old Hanoverian gelding. Cranial is to the left.

C6 was considered normal if the two distinctive ventral laminae of the transverse processes could be identified (Figure 2). An asymmetric appearance of the transverse processes, resulting from unilateral or bilateral absence of the caudal ventral laminae, led to classification as ECCMV (Figure 3a,b). Horses with radiographs that did not enable a clear assessment of C6 due to poor image quality were excluded from the study.

**Figure 4:**
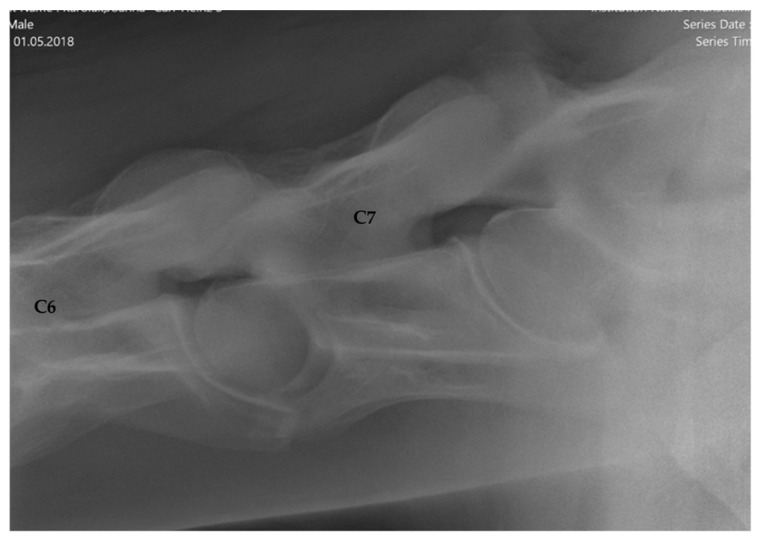
Latero-lateral radiographic image of the sixth to seventh (C6–C7) cervical articular process joint without abnormal findings (grade 0) of a 6-year-old Holsteiner gelding. Cranial is to the left.

**Figure 5:**
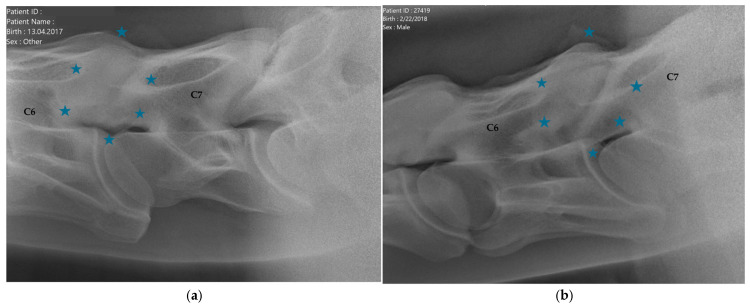
(**a**) Latero-lateral radiographic image of the sixth to seventh (C6–C7) cervical articular process joint with mild enlargement and sclerosis, with intervertebral foramen slightly obscured by new bone formation, encircled with blue asterisks (grade 1), of a 7-year-old Hanoverian gelding. Cranial is to the left. (**b**) Latero-lateral radiographic image of the sixth to seventh (C6–C7) cervical articular process joint with moderate to severe enlargement and sclerosis, significant reduction of the intervertebral foramen, encircled with blue asterisks (grade 2), of a 6-year-old Hanoverian gelding. Cranial is to the left.

The articular process joints (APJ) of C6/C7 were evaluated and with respect to signs of modelling/osteoarthritis assigned to three different groups, based on the APJ osteoarthritis (OA) categorization of other authors [15,20] (Figure 4 and Figure 5a,b):

- Group 1 = normal: no periarticular new bone formation at ventral margins of APJ, intervertebral foramina clearly visible.
- Group 2 = mild APM: minimal/equivocal enlargement and sclerosis of APJ with mild osteophytosis ventrally, intervertebral foramina open/slightly obscured by new bone formation.
- Group 3 = moderate/severe APM: clear enlargement and sclerosis of APJ with moderate osteophytosis ventrally, significant reduction/loss of intervertebral foramina.

#### 2.1.3. Correlation of ECCMV and Sex

The association between the prevalence of ECCMV and sex was determined using Fisher’s exact test due to small cell counts and unequal group sizes. Significance level was set at a *p*-value of <0.05.

#### 2.1.4. Correlation of ECCMV and APM

To investigate a potential association between ECCMV and APM, data were summarized in 2 × 2 contingency tables comparing the distribution of APM across horses with and without ECCMV. ECCMV was treated as a binary variable (present vs. absent). Although APM was initially graded into three categories (normal, mild, moderate/severe), categories were collapsed for statistical testing into a dichotomous outcome (normal vs. any degree of modelling) to ensure adequate cell sizes.

Differences between groups were primarily evaluated using the Chi-squared test with Yates’ continuity correction. When expected cell frequencies were <5, Fisher’s exact test (two-tailed) was additionally applied. Effect size was expressed as odds ratios (OR) derived from the contingency tables. Statistical significance was set at *p* < 0.05. All analyses were performed using SPSS Statistics Version 25 (IBM Corp., Armonk, NY, USA).

#### 2.1.5. Tracing of Competition Performances

The horses’ individual performance data were collected following records obtained from the German National Equestrian Federation (FN), the official provider of German sport and breeding results. Registered placings in dressage and jumping on a national level as well as the lifetime earnings of each horse (up until September 2024), identified by Universal Equine Life Number (UELN), were documented.

Athletic performance was assessed by categorizing horses into three levels according to their highest achieved rank in the national FN database:

- Young horse tests (classes A and L, jumping 0.95–1.15 m, at least 5–10 or >10 placings).
- Medium level (class M, jumping 1.20–1.35 m, ≤10 or >10 placings).
- Advanced level (class S to Grand Prix, jumping ≥ 1.40 m, any number of placings).

To evaluate a potential association between the presence of ECCMV and performance outcomes in the respective levels (young horse, medium, advanced), Fisher’s exact test was applied due to small sample sizes. Odds ratios (OR) and 95% confidence intervals (CI) were calculated to quantify the strength of associations. Statistical significance was set at *p* < 0.05.

## 3. Results

### 3.1. Study Population

A total of 200 horses met the inclusion criteria for this study: 139 males, including 127 geldings (63.5%), 12 stallions (6%) and 61 females (30.5%). At the time of the pre-purchase examination, the median age was 5 (mean 6.18, interquartile range (IQR) 3). The predominant breed was Hanoverian (131 horses, 65.5%), followed by Oldenburg (27 horses, 13.5%) and Holsteiner (22 horses, 11%). Six German riding ponies and Royal Dutch Sport Horses (KWPN) were presented, respectively (3%). Twelve horses belonged to warmblood breeds that occurred only once or twice in this population (<3%).

Twelve horses were excluded in advance because they did not pass the pre-purchase examination due to lameness. Five horses were disqualified due to inadequate radiographic image quality.

### 3.2. Prevalence of ECCMV and Articular Process Joint Modelling (APM)

C6 was classified as normal with two distinct ventral laminae in 140 horses (70%, Figure 1). In 60 horses (30%), ECCMV with a unilateral or bilateral, complete or partial absent ventral lamina was found (Figure 3a,b).

With respect to sex, ECCMV was identified in 36 of 139 male horses (25.9%) and in 24 of 61 female horses (39.3%). Unilateral absence of the caudal ventral lamina of C6 was observed in 22 males (15.8%) and 13 females (21.3%), whereas bilateral absence was present in 14 males (10.1%) and 11 females (18.0%). There was a statistical trend (Fisher’s exact test *p* = 0.057, OR = 1.85) for females to show a higher prevalence of both unilateral and bilateral laminar absence. The distribution of unilateral and bilateral ECCMV according to sex is summarized in Table 1.

### 3.3. Prevalence of Articular Process Joint Modelling (APM)

The APJ of C6/C7 was considered normal in 135 horses (67.5%, Figure 3), whereas signs of modelling (APM) were present in 65 horses (32.5%). Mild radiographic changes were evident in 53 horses (26.5%), and moderate to severe osteoarthritic findings in 12 horses (6%). For exemplary images, see Figure 5a,b.

The group of horses with a normal C6 appearance (*n* = 140) comprised 89 (63.6%) normal APJs, 40 (28.6%) with mild (grade 1) APM findings and 11 (7.9%) with moderate/severe (grade 2) OA characteristics. In total, 51 horses (36.5%) within this group exhibited APM.

Within the ECCMV group (*n* = 60), 46 (76.7%) APJ were regarded as normal; 13 (21.7%) showed mild signs and 1 (1.7%) showed moderate/severe signs of APM, adding up to 14 (23.4%) altered joints in total.

**Figure 6:**
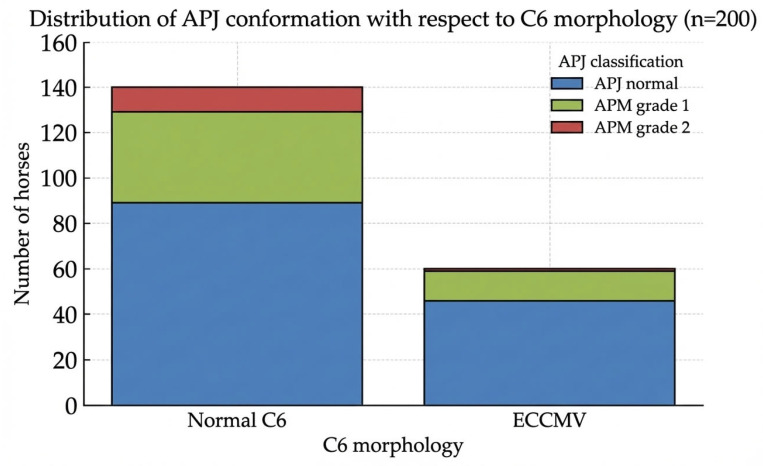
Distribution of articular process joint (APJ) conformation in warmblood sport horses with normal C6 morphology and equine caudal cervical morphologic variation (ECCMV) (*n* = 200). APJ changes were classified as normal, mild modelling (grade 1), or moderate to severe modelling (grade 2).

Figure 6 (and Table A1 in Appendix A) provides an overview of the distribution of APJ conformation with respect to ECCMV.

Although fewer horses with ECCMV exhibited altered APJs, especially grade 2 modelling, this correlation could not be proven statistically: The Chi-squared test with Yates’ correction yielded a test statistic of χ^2^ = 0.92 (*p* = 0.339), indicating no significant association between ECCMV and APM after dichotomization of APJ categories. Although horses with ECCMV were less likely to exhibit radiographic APM compared to those with normal C6 morphology, Fisher’s exact test (two-tailed) similarly showed no significant difference (*p* = 0.323) with an odds ratio of 0.68.

### 3.4. Inter-Observer Agreement on ECCMV Radiographic Findings

Inter-observer agreement for the detection of ECCMV was assessed using Cohen’s kappa (κ). The analysis revealed an almost perfect agreement between the ECVSMR/ACVSMR and ECVDI diplomates, with a kappa value of 0.85 (observed agreement: 94%). Discrepancies were noted in only 12/200 cases (6%), which were subsequently resolved through a consensus reassessment.

### 3.5. Competition Performances

To evaluate the impact of ECCMV on athletic outcome, horses were categorized into three performance levels based on their highest achievement in dressage and show jumping: (1) young horse tests (classes A and L, at least 5–10 or >10 placings), (2) medium level (class M, ≤10 and >10 placings), and (3) advanced level (class S to Grand Prix, any number of placings).

Of the 200 horses, 71 (35.5%) had achieved at least five placings in young horse tests at the time this study was conducted. Within this group, no significant difference was observed between horses with normal C6 morphology (54/140) and those with ECCMV (17/60) (38.6% vs. 28.3%, *p* = 0.16). Specifically, 15.0% of normal C6 horses (21/140) and 13.3% of ECCMV horses (8/60) achieved 5–10 placings (*p* = 0.83, OR = 1.15), while 23.6% (33/140) and 15.0% (9/60), respectively, achieved more than 10 placings (*p* = 0.19, OR = 1.75).

At the medium level (class M, jumping 1.20–1.35m), 32 horses (16.0%) were successfully placed overall. The amount of placings at this level was similar between both groups, with 15.0% for normal C6 (21/140) and 18.3% for ECCMV (11/60) horses (*p* = 0.54, OR = 0.79). More precisely, up to 10 placings were achieved by 13 horses without (13/140 = 9.3%) and 9 horses with ECCMV (9/60 = 15%). More than 10 placings at this level were earned by 8 horses without (8/140 = 5.7%) and 2 with ECCMV (2/60 = 3.3%).

**Figure 7:**
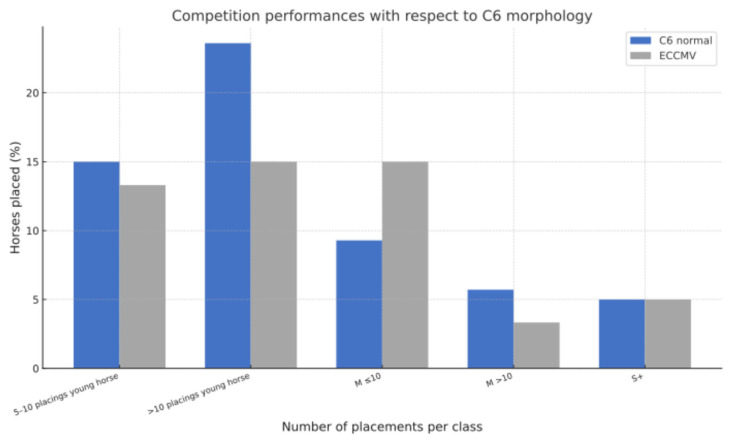
Competition performance outcome of warmblood sport horses with normal C6 morphology vs. horses with ECCMV. The bar chart illustrates the percentage of horses achieving specific placements combined in categories such as young horse classes, 5–10 and >10 placings, class M ≤10 placings, class M >10 placings, and placings in class S and above within each C6 morphology group. No significant differences in competition success were observed between horses with normal C6 and those with ECCMV across the evaluated performance classes.

Advanced level success (class S, jumping ≥ 1.40 m and dressage class S and Grand Prix) was recorded for 10 horses (5.0%), including 7 horses with normal C6 (7/140 = 5%) and 3 horses with ECCMV (3/60 = 5%). Due to the small sample size in this highest category, statistical analysis was not performed. Overall, Fisher’s exact test revealed no significant association between the presence of ECCMV and any of the defined performance outcomes (all *p* > 0.05). A detailed breakdown of the underlying numbers for each category is provided in Appendix A Table A2. Figure 7 displays a graphical illustration of placements of horses with and without ECCMV at the respective levels.

Concerning lifetime earnings, 77 of 140 horses (55%) with a normal C6 generated prize money by placing in a competition. The earned sums ranged from 9 EUR to 40,801.50 EUR. A median of 239 EUR per horse was earned (mean = 1183.34 EUR, IQR = 526 EUR).

Among horses with ECCMV, 30 of 60 (50%) had achieved placements at the time of data collection, with lifetime earnings ranging from 6 EUR to 4.820 EUR. A median of 194.50 EUR was collected per horse (mean = 618.98 EUR, IQR = 942.75 EUR).

## 4. Discussion

### 4.1. Clinical Significance and Etiology of ECCMV

In the present study, ECCMV was identified in approximately one-third of clinically sound warmblood sport horses and was not associated with impaired competition performance or an increased prevalence of APM. These findings support the interpretation of ECCMV as an anatomical variation rather than an inherently pathological condition, particularly in non-clinical populations.

The etiology of ECCMV is considered congenital [5,7,9]. However, sport-specific biomechanical demands influence how such congenital variations interact with adjacent structures like the musculus longus colli over time. This paired perivertebral muscle acts as an important stabilizer, rotator and flexor of the cervicothoracic junction and its thoracic portion inserts on the ventral lamina of C6 [10]. May-Davis et al. suppose that the absence of this attachment site in horses with ECCMV may alter muscle function and force transmission, potentially affecting cervical and postural stability, not least because of this muscle’s rich proprioceptive innervation [5,9]. To the author’s knowledge, this assumption has not been substantiated by longitudinal studies yet.

More recent prospective research by Dyson et al. [2,22] on the contrary suggests that these altered biomechanical forces do not necessarily translate into clinical disease. The clinical relevance of such morphologic variations might be overinterpreted unless evaluated together with the horses’ individual signs when thoroughly examined.

### 4.2. ECCMV with Respect to Age, Sex and Breed

Age-related effects on the radiographic appearance of ECCMV are considered unlikely, as these variations are regarded congenital. However, secondary adaptive or degenerative changes in adjacent structures may become more apparent with increasing age. The relatively young median age of the present population may therefore limit the detection of long-term biomechanical consequences.

Breed-related differences in ECCMV prevalence have been reported. In a postmortem study by May-Davis et al. [9], C6 variations were identified in 23 of the 123 horses examined (18.7%). ECCMV was predominantly found in thoroughbreds (*n* = 19/50) and thoroughbred derivatives (*n* = 3/3), while only one nondescript bred horse (*n* = 1/15) and no mixed-breed horses (*n* = 0/55) were affected. Notably, 22 of the 23 affected horses were of thoroughbred lineage. This prevalence of 38% (19/50) is among the highest reported rates for a single breed. Warmblood breeds appear more frequently affected as well. A comparative study of breed differences in the anatomical configuration of the whole equine vertebral column, examined postmortem via computed tomography, yielded an ECCMV prevalence rate of 43% in warmbloods (and 6% in Shetland ponies) [23]. It must be considered that the superior imaging capabilities of CT may have resulted in fewer false negative diagnoses compared to conventional radiographic examinations. However, another postmortem CT study investigated 78 horses of different breeds and indicated a prevalence of 33.3% [1]. A radiographic study on 100 horses found an overall prevalence of 24% but a significantly higher occurrence of ECCMV in warmblood breeds (19/55 = 34.5%) [11]. Dyson et al. conducted a cross-sectional study on the association of radiographic ECCMV with clinical signs in 223 warmbloods and ascertained a considerably lower prevalence of 24.2% [2]. Another retrospective observational radiographic study on 135 cases, comprising warmbloods, thoroughbreds and quarter horses found an overall prevalence of 20% but 28% (17/60) in the respective warmblood breeds [3]. Santinelli et al. applied the same study design to investigate ECCMV in a mixed-breed population of 270 horses, comprising 138 warmbloods, and detected an overall prevalence of only 13%, comparable to 15.2% of the examined warmbloods’ radiographs [8]. A large but, again, retrospective radiographic case–control study on 377 warmblood horses identified ECCMV in overall 28.6%, including 23.7% cases and 38% controls [20]. Beccati et al. investigated radiographic findings in mixed breeds with clinical signs and found ECCMV in 24/166 = 20.7% [12]. Espinosa-Mur et al. conducted a study on radiographic cervical OA in 104 warmblood show jumpers and determined an ECCMV prevalence of 23/104 = 22.1% [18]. Two retrospective studies focused on Dutch warmbloods, again using radiography as image modality. Veraa et al. evaluated measurements of the intervertebral disc space width in horses aged 1–18 months and found C6 morphologic variations in 10/28 = 35.7% [24], whereas Crijns et al. established a grading system for APJ OA and diagnosed ECCMV findings in 168/664 = 25.3% of mature Dutch warmbloods [25].

Taken together, these studies demonstrate a substantial variability in reported ECCMV prevalence rates (13–43%) across breeds and study populations, with warmblood and occasionally thoroughbred lineages showing higher rates than other breeds. Reported differences are likely influenced by a combination of breed composition, study design and imaging modality applied, as postmortem and CT-based studies provide a higher sensitivity. Therefore, prevalence data should be interpreted in the context of methodological differences. A comparative overview of study design, study populations, breed, imaging modalities and reported prevalence rates is provided in Table A3.

With respect to sex, ECCMV was identified in 25.9% of male and in 39.3% of female horses. While some authors did not find a statistically significant association with sex [3,11], Santinelli et al. retrospectively investigated radiographs of the cervical spine of 270 horses and did find sex-related differences: 21% (22/106) of females vs. 8% (14/164) of males exhibited a partial or complete transposition of the ventral process of C6 onto C7 [8]. This corresponds well with the current study’s findings, although the higher prevalence in females narrowly did not reach statistical significance. Further cross-sectional studies with larger cohorts are warranted to affirm this association.

### 4.3. Prevalence of ECCMV

The prevalence of ECCMV (30%) in the current research corresponds well with other studies investigating anatomical variants of C6 in warmbloods. As outlined above, current prevalence data range from 13% to 24% in a mixed-breed population [8,11], over 24% to 28% in warmbloods [2,3] and up to 38% in thoroughbreds [9]. Postmortem CT examinations of the cervical spine by Veraa et al. (2016) [1] revealed that 33.3% (26/78) of horses exhibited conformational variations of C6 in accordance with ECCMV. A subsequent retrospective case–control study involving 377 horses by Veraa et al. (2020) showed that 28.6% overall, including 23.7% of cases (58/245) and 38% (50/132) of controls, had morphologic changes of C6 on radiographs [20]. In another retrospective study on 100 horses by DeRouen et al. (2016) [11], warmblood breeds accounted for almost 80% of horses with anatomical variants of C6, with a prevalence of 35% (19/55) amongst this breed.

Due to the limited number of horses with ECCMV and to avoid division into even smaller groups, no further distinction was made regarding the absence of one or both ventral laminae except with regard to sex. All horses included in this study were presented for pre-purchase examinations and underwent a highly standardized work-up by one highly experienced veterinarian (Dipl. ECVSMR/ACVSMR).

The high prevalence of ECCMV in the investigated, clinically sound sport horse population and the results of two recent studies where congenital variants of C6 occurred more frequently in controls compared with cases [2,20], support the hypothesis formulated by Veraa et al. [20] that these morphologic changes might even indicate a beneficial adaption in warmbloods. Alterations in the morphology of C6 could influence the distribution of biomechanical forces within the caudal cervical spine, potentially reducing mechanical stress on adjacent structures such as the APJ. However, the present study was not designed to assess biomechanical function; therefore, any presumed beneficial effect remains speculative. Nonetheless, the absence of negative associations with clinical soundness or competition performances in the present research further supports the interpretation of ECCMV as an anatomical variation rather than a performance-limiting malformation. Moreover, this study determined a prevalence of 30% which reinforces the assumption that warmblood breeds are more frequently affected [24].

### 4.4. Prevalence of APM

In our own investigations, the APJ of C6/C7 was considered normal in 67.5% of horses (135/200). Mild and moderate to severe osteoarthritic signs were found in 26.5% (53/200) and 6% (12/200), respectively. One reason for the relatively low prevalence of APM in this study may be the young age of the study population, with a median of 5 years. A study on 104 actively competing show jumpers demonstrated that higher grades of OA at C6/C7 were associated with a higher competing level. At the age of 5 years, however, horses are still limited to low-impact young horse classes. The comparatively low prevalence of APM might also be attributed to the radiographic technique used in this study, which was restricted to latero-lateral views and therefore may have limited the detection of morphological changes in the APJ.

The articular processes connect the adjacent vertebrae and facilitate movement dependent on the alignment of their joint surfaces, which is predominantly dorsoventral flexion and extension in the caudal cervical spine [13]. The authors of this study decided to use the term ‘articular process joint modelling’ alongside osteoarthritis to underline that a certain degree of enlargement or remodelling might be a physiological adaption to biomechanical stress like pronounced extension of the cervicothoracic region, and not necessarily be associated with degenerative joint disease [2,14]. Nevertheless, the literature on APM regarding prevalence and clinical implications is conflicting. In the current study, 32.5% showed radiographic signs of APM without corresponding clinical observations. In a case–control study on the relationship of cervical radiographic variations and clinical presentations by Veera et al., only 17.4% (23/109) of asymptomatic control horses had imaging findings indicating OA [20]. On the contrary, in a population of 111 sound and actively performing warmblood jumpers, radiographic signs of mild OA were present in 42.3% (44/111), and signs of moderate to severe OA were present in 25% (26/111) of horses. Several studies indicate that radiographic changes in the cervical facet joints are present in at least 50% of clinically normal horses [21,26]. A more recent case–control comparison revealed that 96.7% (29/30) of controls showed APM on radiographs, although the grade of modelling at C5/C6 and C6/C7 was significantly higher in cases compared with controls [19]. The same applies to latest study results by Dyson et al. (2025) [2], where cases (31/96) were more likely to have severe modelling compared with control horses (17/127).

### 4.5. Association Between ECCMV and APM

In the latest study just cited, there was no difference between the degree or the prevalence of APM and the presence or absence of congenital variants of C6 [2]. The same applies for a case–control study on 116 horses by Beccati et al., where the authors conclude that ECCMV does not predispose the horse to the development of more severe radiographic changes in the caudal APJs [12]. This corresponds well with the current study’s findings, since a statistically significant link between ECCMV and APM could not be demonstrated either. Interestingly, fewer horses with ECCMV exhibited altered APJs in both studies: Dyson et al. found that 87% of horses with ECCMV (47/54) but 91.7% of horses with a normal C6 (155/169) showed signs of cervical facet modelling [2]. Within the present study population, only 23.3% of horses with (14/60) but 36.4% of horses without ECCMV (51/140) showed radiological signs of APM.

However, a considerably larger study population would be required to further investigate or prove a potential negative correlation of ECCMV with APM. To date, the hypothesis proposed by the authors of this study about an association of these two conditions could not be proven statistically.

### 4.6. Inter-Observer Agreement

The inter-observer agreement on ECCMV as a radiographic finding was excellent, since only 12/200 (6%) radiographs had to be reassessed. This suggests that latero-lateral radiographs are usable to diagnose morphological variations of C6. Numerous other research groups investigating the caudal cervical area relied exclusively on this approach [3,8,11,12,20]. Nonetheless, computed tomography or postmortem examinations would be required to validate the present diagnoses.

### 4.7. Competition Performances

To the authors’ knowledge, there are no studies on competition performances of horses with and without ECCMV. Our research demonstrates that there is no significant difference in athletic performance, measured by the number and level of placements, between horses with a normal C6 morphology and those with ECCMV. Concerning lifetime earnings, horses with and without ECCMV earned similar median amounts of prize money whereas the mean value is nearly twice as high in the non-ECCMV group due to individual extraordinarily successful horses.

It must be taken into account that the sporting success of a horse largely depends on extrinsic factors like the skill level of the rider, the condition and fitness of the horse (irrespective of the cervical spine), the quality of training and general competition preparations and lastly the competitors in the respective classes.

Furthermore, our study refers solely to competition results on a national level, although a large proportion of the horses presented for pre-purchase examinations were exported to the United States of America. Tracking show results there is considerably more difficult and unreliable.

With a median age of 5 years, the study population was still young, and since the minimum age for young horse tests in Germany is 4 years, most horses had only been able to perform in one or two competition seasons at the time this study was conducted.

Regarding lifetime earnings on a closer inspection, a slightly higher proportion of horses with normal C6 morphology (55%) earned prize money compared to those with ECCMV (50%). The range of earnings was substantially wider in the normal C6 group (9 EUR–40,801.50 EUR) than in the ECCMV group (6 EUR–4.820 EUR), and both median (239 EUR vs. 194.50 EUR) and mean (1183.34 EUR vs. 618.98 EUR) values were higher in horses without ECCMV. The large interquartile ranges, particularly in the normal C6 group, indicate that a few high-performing individuals substantially influenced the mean, highlighting that median values better represent overall performance. The likelihood of earning prize money is still broadly comparable between both groups, although horses without ECCMV tended to achieve higher earnings.

Nevertheless, what can be deduced from these results is that horses with ECCMV can successfully be placed in dressage and jumping classes on any national level. The hypothesis regarding comparable tournament success of horses with and without ECCMV could therefore be confirmed.

Overall, the absence of both clinical signs and performance limitations in horses with ECCMV in this study population suggests that the presence of ECCMV alone should be interpreted cautiously during pre-purchase examinations and should not automatically be equated with a negative prognosis.

### 4.8. Limitations

#### 4.8.1. Diagnostic Imaging

Limitations that should be considered next to the retrospective observational design of this study and the limited number of cases concern diagnostic imaging, since it was restricted to the acquisition of latero-lateral radiographs to assess the cervical spine.

Interpretation of latero-lateral cervical radiographs should take the position of the X-ray beam into account. Because of beam divergence and the complex three-dimensional morphology of the cervical vertebrae, a projection that is simultaneously perpendicular to both the ventral lamina and the APJ cannot be achieved. Consequently, standard lateral views may exaggerate the apparent size of the APJ due to projectional effects [13]. Oblique radiographs can reduce this distortion, and computed tomography (CT) could have detected cervical vertebral changes at the ventral aspect of C6 as well as of the APJ more accurately. Among available imaging modalities, latero-lateral radiography is still the most commonly used technique for screening the caudal cervical region in a standing horse and represents the standard imaging approach during pre-purchase examinations. The primary benefits are its simplicity, quick feasibility and accessibility both in hospital environments and ambulatory practice. Unlike CT, requiring general anesthesia for complete visualization of the caudal neck, radiography is barely stressful for the horse and can frequently be performed with minimal or even no sedation. Furthermore, modern digital radiography systems provide excellent image quality and allow for immediate on-site evaluation, facilitating a rapid diagnostic workflow.

Nevertheless, computed tomography remains the gold standard for depicting complex anatomy, and a clear disadvantage of two-dimensional radiographic projections is the superimposition of surrounding structures, implicating an overall reduced sensitivity for subtle morphological changes like partial laminar remnants or transpositions.

A large retrospective case–control study by Veraa et al. investigating ECCMV in 377 horses was based solely on latero-lateral radiographic projections [20]. The same applies for another retrospective study on 135 horses presented for cervical spine radiography where ECCMV was part of the investigations [3]. Research by Santinelli et al., focusing on radiographic morphologic variations of the caudal cervical and the first thoracic vertebra in 270 horses, is based on latero-lateral images as well [8]. None of these three studies incorporated statistical analyses of inter-observer agreement on radiographic findings. This represents a methodological gap that the present work addresses, thereby strengthening the reliability of our data.

Withers et al. stated that the transposition of the ventral lamina of C6 onto C7 may be less visible on oblique views but more clearly seen on latero-lateral images, thus emphasizing the informative value of these projections [7,13].

Nonetheless, several studies rely on additional oblique views to assess the caudal cervical spine and, in particular, the ventral lamina of C6 [2,4,6,7], but since they were not available in most of the horses included in this study and due to its retrospective nature, the authors relied on latero-lateral image capturing. The assignment of two highly qualified independent reviewers and the high interobserver agreement in the present study further supports the reliability of this imaging modality for identifying ECCMV when evaluated by experienced observers.

#### 4.8.2. Gait Analysis

Another limitation of this study concerns the orthopedic examination: It was carried out without the use of artificial intelligence-assisted objective gait analysis tools, since they were not an established part of pre-purchase protocols at that time. Therefore, it cannot be ruled out that horses with a very subtle lameness are included.

#### 4.8.3. Competition Outcome

Since this is no longitudinal study, competition performances were evaluated over a specific period only and may look considerably different at another point in time. Extrinsic factors like the rider’s expertise were not investigated but clearly influence performance outcomes.

## 5. Conclusions

The prevalence of ECCMV amounted to 30% in this warmblood sport horse population, consistent with previous reports. Within the ECCMV subtypes assessed, no negative association was identified between these anatomical variations and performance outcomes in dressage or show jumping.

Radiographic signs of APM were overall present in 32.5% of cases. There was no significant association between the presence of ECCMV and APM, but horses with ECCMV were less likely to exhibit radiographic changes in the adjacent APJ compared to those with a normal C6 morphology (23.3% vs. 36.4%).

Further studies differentiating ECCMV subtypes and incorporating longitudinal and biomechanical data are required to clarify their functional significance. To date, it remains hypothetical if certain ECCMV phenotypes represent a compensatory biomechanical adaptation rather than a pathological condition, potentially influencing load distribution within the cervical spine and thereby contributing to a presumed mitigation of APM.

## Figures and Tables

**Table 1:** Uni- or bilateral absence of the caudal part of the ventral lamina of C6 with respect to sex. The relative number of affected horses per sex is indicated in parentheses.

ECCMV	Male	Female	Total
unilateral	22 (15.8%)	13 (21.3%)	35
bilateral	14 (10.1%)	11 (18%)	25
total	36 (25.9%)	24 (39.3%)	60

## Data Availability

The raw data supporting the conclusions of this article will be made available by the authors on request.

## Abbreviations

The following abbreviations are used in this manuscript:

C6	Sixth cervical vertebra
C7	Seventh cervical vertebra
CI	Confidence interval
CT	Computed tomography
ECCMV	Equine caudal cervical morphologic variations
OA	Osteoarthritis
OR	Odds ratio
Dipl.	Diplomate
ECVSMR	European College of Veterinary Sports Medicine and Rehabilitation
ACVSMR	American College of Veterinary Sports Medicine and Rehabilitation
ECVDI	European College of Veterinary Diagnostic Imaging
GEVA	German Equine Veterinary Association
GPM	Gesellschaft für Pferdemedizin
k	Kilovoltage
mAs	Milliampere-seconds
FN	Fédération Equestre (German National Equestrian Federation)
UELN	Universal Equine Life Number
KWPN	Koninklijk Warmbloed Paardenstamboek Nederland (Dutch Warmblood)
IQR	Interquartile Range
APJ	Articular process joint
APM	Articular process joint modelling

## Appendix A

**Table A1:** Distribution of articular process joint (APJ) conformation in warmblood sport horses with normal C6 morphology and equine caudal cervical morphologic variation (ECCMV) (*n* = 200). APJ changes were classified as normal, mild modelling (grade 1), or moderate to severe modelling (grade 2).

C6 Morphology	APJ Normal	APM Grade 1	APM Grade 2	Total
C6 normal	89	40	11	140
	(63.6%)	(28.6%)	(7.9%)	
ECCMV	46	13	1	60
	(76.7%)	(21.7%)	(1.7%)	
Total	135	53	12	200

**Table A2:** Competition performance outcomes of warmblood sport horses with normal C6 morphology vs. horses with ECCMV, categorized in three performance levels (young horse, medium, advanced).

Performance Category	C6 Normal (*n* = 140)	ECCMV (*n* = 60)	Total (*n* = 200)
Young horse tests 5 to 10 placings >10 placings	54 (38.6%)	17 (28.3%)	71 (35.5%)
	21 (15%) 33 (23.6%)	8 (13.3%) 9 (15%)	29 (14.5%) 42 (21%)
Medium level/class M 1 to 10 placings >10 placings	21 (15%) 13 (9.3%)	11 (18.3%) 9 (15%)	32 (16%) 22 (11%)
	8 (5.7%)	2 (3.3%)	10 (5%)
Advanced level/class S/GP	7 (5%)	3 (5%)	10 (5%)

**Table A3:** Reported ECCMV prevalences according to study design, study population size, breed and imaging modality. WB = warmblood, TB = thoroughbred, QH = quarter horse.

Authors	Study Design	Population (n)	Breed	Imaging Modality	Prevalence
May-Davis et al. [9]	cross-sectional	123	TB, TB-derived, mixed	Postmortem dissection	18.7% overall, 38% TB
Veraa et al. [1]	retrospective	78	mixed	Postmortem CT	overall 33.3%
DeRouen et al. [11]	retrospective	100	mixed	Radiography	24% overall, 34.5% WB
Dyson et al. [2]	cross-sectional	223	WB	Radiography	24.2%
Henderson et al. [3]	retrospective	135	WB, TB, QH	Radiography	20% overall, 28% WB
Santinelli [8]	retrospective	270	mixed	Radiography	13.3% overall, 15.2% WB
Veraa et al. [20]	cross-sectional	377	WB	Radiography	28.6%
Espinosa-Mur et al. [18]	retrospective	104	WB jumpers	Radiography	22.1%
Veraa et al. [24]	prospective	28	Dutch WB yearlings	Radiography	35.7%
Crijns et al. [25]	retrospective	664	Dutch WB	Radiography	25.3%
Beccati et al. [12]	retrospective	166	mixed	Radiography	20.7%
Spoormakers et al. [23]	cross-sectional	77	mixed	Postmortem CT	43% WB
Present study	retrospective	200	WB	Radiography	30%
